# Electrophysiological measurement of the effect of inter-stimulus competition on early cortical stages of human vision

**DOI:** 10.1016/j.neuroimage.2014.10.033

**Published:** 2015-01-15

**Authors:** Claire E. Miller, Kimron L. Shapiro, Steven J. Luck

**Affiliations:** a School of Psychology, Bangor University, Bangor, LL57 2AS, UK; b School of Psychology, University of Birmingham, Birmingham, B15 2TT, UK; c Center for Mind and Brain, University of California, Davis, CA 95618, USA

**Keywords:** Event-related potential, Vision, Competition, C1, P1

## Abstract

Competition between inputs in early visual cortex has been established as a key determinant in perception through decades of animal single cell and human fMRI research. We developed a novel ERP paradigm allowing this competition to be studied in humans, affording an opportunity to gain further insight into how competition is reflected at the neural level. Checkerboard stimuli were presented to elicit C1 (indexing processing in V1), C2 (hypothesized to reflect V1 after extrastriate feedback), and P1 (extrastriate) components. Stimuli were presented in three randomized conditions: single stimulus, near proximity pairs and far proximity pairs. Importantly, near stimuli (0.16° visual angle apart) were positioned to compete in primary visual cortex, whereas far stimuli (2° apart) were positioned to compete in extrastriate visual areas.

As predicted, the degree and spatial range of competition increased from the C1 component to the C2 and P1 components. Specifically, competitive interactions in C1 amplitude were modest and present only for near-proximity pairs, whereas substantial competition was present for the P1, even for far-proximity pairs. To our knowledge, this is the first study to measure how competition unfolds over time in human visual cortex. Importantly, this method provides an empirical means of measuring competitive interactions at specific stages of visual processing, rendering it possible to rigorously test predictions about the effects of competition on perception, attention, and working memory.

## Highlights

• Novel paradigm measuring how competition unfolds over time in human visual cortex.
• Degree and spatial range of competition increased over the first 150 ms poststimulus.
• C1 interactions for near-proximity pairs; large P1 interactions even at far-proximity.

## Introduction

Objects in the external world are constantly competing for representation in the human brain at different scales and in many different parts of the cortex. The resolution of this competition is a vital mechanism that serves to prevent information overload by prioritizing currently relevant information, as described in the highly influential theory of biased competition (Desimone and Duncan, 1995). As an example, competition between visual stimuli through lateral inhibition in the retina (e.g. Hartline et al., 1956, Alpern and David, 1959) has been shown to aid contrast and contour perception by sharpening visual input, and is also implicated in the organization of center-surround receptive fields in bipolar and ganglion cells (Werblin, 1974, Cook and McReynolds, 1998). Moreover, in visual cortex, recent studies suggest that competitive interactions are stronger between stimuli presented within the same visual receptive field (RF) than between stimuli presented in different RFs (Kastner et al., 1998, Reynolds et al., 1999).

Reynolds et al. (1999) demonstrated the presence of competitive interactions between stimuli within RFs using single cell recordings in primates. When an unattended stimulus pair was presented simultaneously within a single V4 RF, the firing rate was not simply the sum of the firing rates for each stimulus presented alone but was instead near the average of the firing rates for the individual stimuli. Further, when one stimulus of a pair was attended, the features of this stimulus entirely determined the cell’s firing rate. Similarly, Luck et al. (1997) found that attention modulated V2 and V4 firing rates only when both the attended and ignored stimuli were inside the neuron’s RF (and thus were in competition for control over that neuron). Moreover, when both stimuli were inside the RF, the attention effect was reduced when the stimuli were presented sequentially rather than simultaneously, presumably because sequential presentation reduces competition between the stimuli (see below). Other studies have also found large single-unit attention effects when both attended and ignored stimuli were simultaneously presented inside the neuron’s RF (Treue and Maunsell, 1996, Moran and Desimone, 1985). It is important to note that single cell methods have been unable to investigate competitive interactions between two stimuli within the same V1 RF, due to small RF size.

In an fMRI study in humans, Kastner et al. (1998) presented the same four stimuli either simultaneously or sequentially in the periphery, while participants performed a task at fixation. They found decreased BOLD activity in response to simultaneous relative to sequential stimuli in areas V1, V2, V4 and TEO, with the difference between the two conditions increasing with RF size. This outcome is consistent with strong competition between stimuli that were presented simultaneously within the same RF, with more items falling within a single RF in areas with large RFs than in areas such as V1 that have small RFs (see also Kastner et al., 2001).

Studies of lateral masking and crowding (e.g., Loomis, 1978) also suggest that stimulus proximity reduces discriminability. In an early study, Flom et al. (1963) found that flanking black bars reduced participants' ability to make a judgment about peripherally presented Landolt Cs, with the interaction reduced as the distance between them increased. Flom et al. suggested this interaction was due to large receptive field sizes in peripheral vision. Indeed, a later study by Levi et al. (2002) suggests that crowding occurs more strongly for peripheral than foveal stimuli. However, it is difficult to determine from these studies whether the observed decrements in performance reflect interactions in feedforward or feedback processing.

Similarly, although Kastner et al. (1998) found evidence of inter-stimulus competition as early as V1, the poor temporal resolution of the hemodynamic response made it impossible to determine whether the V1 competition they observed reflected competition within V1 during feedforward processing or competition at later stages feeding back to V1. On the other hand, the excellent temporal resolution of the event-related potential (ERP) technique makes it ideal for assessing the different stages in visual processing at which competition can exert its effect in human neural populations and forms the basis of the present investigation.

In the present study, ERPs were used to assess the effects of inter-stimulus competition on early visual ERP components. The components of interest were: C1, thought to originate in primary visual cortex (Clark et al., 1995, Di Russo et al., 2002, Jeffreys and Axford, 1972); C2 (Fortune and Hood, 2003, Kappenman and Luck, 2012), which we suggest to reflect V1 activity after feedback from extrastriate areas; and P1, thought to reflect extrastriate areas, including area V3 and middle-occipital gyrus, and anterior V4v (Di Russo et al., 2002). Our goal was to demonstrate that ERPs can provide a temporally sensitive index of early stimulus competition at varying levels of the early visual system. This is an important first step towards being able to study how competitive interactions in early visual activity depend on bottom-up factors such as stimulus similarity and top-down factors such as attention.

**Fig. 1:**
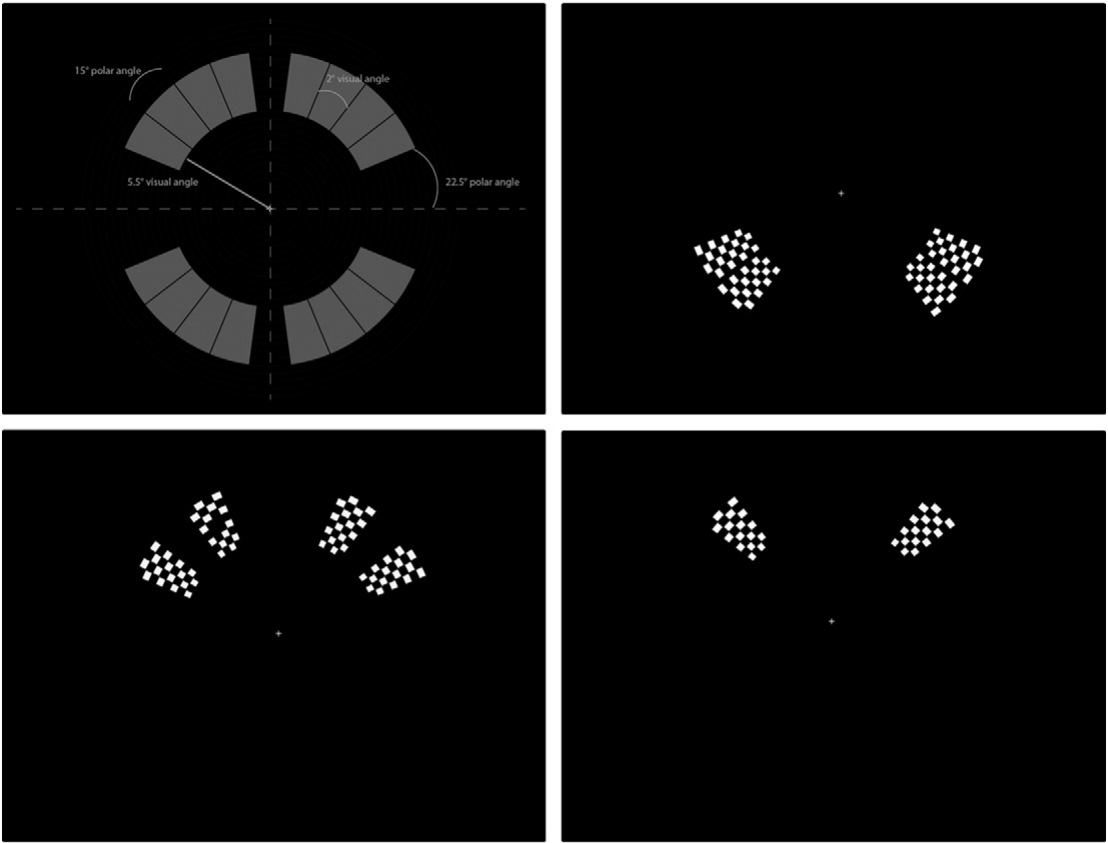
Top left: Locations in which stimuli could be presented (grey segments) with measurements indicating size and position. Top right: Example of experimental screen layout for a dual-near target absent trial. In each trial type, the stimuli could be presented above or below fixation (each occurring on 50% of trials). Bottom left: Dual-far trial, with target present (upper left stimulus). Bottom right: Single stimulus target absent trial.

**Fig. 2:**
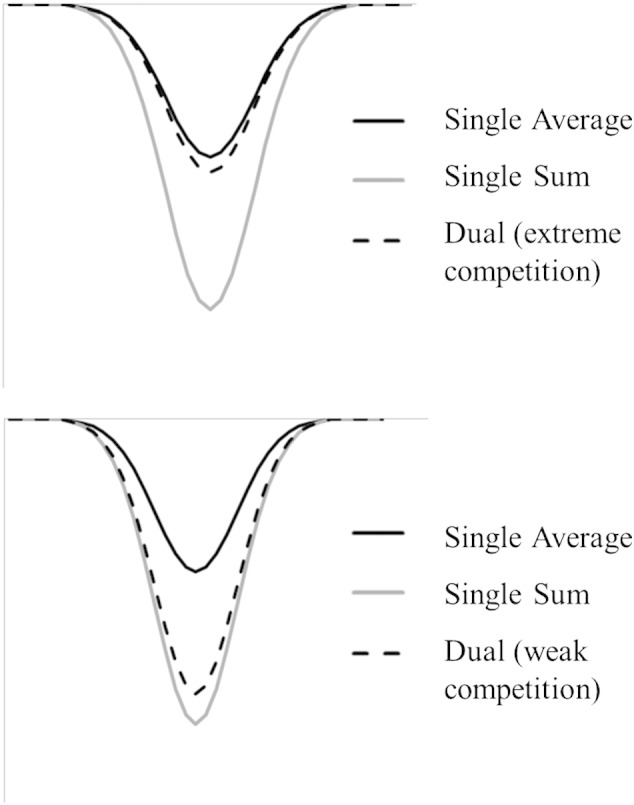
Expected ERP waveforms given weak competition (top panel) and extreme competition (lower panel) between two simultaneous stimuli.

Our experimental approach took advantage of the fact that voltages in the brain directly summate (Nunez and Srinivasan, 2006). Thus, if stimuli presented at two different locations are processed independently, the ERP response to the two stimuli presented simultaneously will be exactly the same as the sum of the ERP responses to the two stimuli presented individually. Consequently, if the observed ERP to two simultaneous stimuli differs from the sum of the ERPs to the individual stimuli at a given point in time, the two stimuli must be interacting with each other at that time. We therefore presented stimuli sequentially at two locations to obtain the responses to the individual stimuli and also presented the stimuli simultaneously at these two locations (see Fig. 1, Fig. 2). This approach has been used extensively in research on binaural interactions between auditory stimuli presented simultaneously to the two ears compared with stimuli presented separately to each ear (see Pratt, 2012 for a review). We also varied the distance between the two locations so that we could test the hypothesis that interactions between stimuli would occur earlier for nearby locations than for more distant locations. Specifically, we predicted that the near stimuli would compete beginning in primary visual cortex, leading to interactions between simultaneous stimuli beginning with the C1 wave. The far stimuli were predicted to compete only in later visual areas, leading to interactions in the P1 wave but not in the C1 wave.

## Method

### Participants

Twenty participants were recruited from the Bangor University participant pool, and each took part in a testing session lasting approximately 2.5 hours in exchange for either course credit or payment of £6/hour. Three were later excluded, two due to the detection of artefacts that exceeded an a priori criterion of > 30% of trials, and one due to a target hit rate of lower than 2 SD below the mean. This resulted in a final sample of N = 17 (ages 18–31, mean age = 21.2, 8 females).

### Stimuli

Stimuli were presented using Presentation experimental control software (Neurobehavioral Systems, Albany, CA) on a 24″ Samsung LCD monitor with a black background (luminance 0.52 cd/m2) and a continuously visible white fixation point. Each display contained two or four small wedge-shaped checkerboards. As shown in Fig. 1, each wedge extended from 5.5 to 9.5° of visual angle from the fixation point and had a width of 15° of polar angle (2° of visual angle in the center of the checkerboard). The checks in center of the checkerboard subtended 0.5° × 0.5° visual angle. The luminance was 0.52 cd/m^2^ for the black checks and 250 cd/m^2^ for the white checks.

Each display contained either a single checkerboard or two simultaneous checkerboards on the left side of fixation, either in the upper or lower half of the screen. To maximize the size of the visual ERP response, stimuli were also presented simultaneously in mirror-image positions in the right visual field (Fu et al., 2010). On 10% of trials, one of the checkerboards was a target (see Fig. 1, bottom left panel) in which two of the central checks were black instead of white. As described below, subjects performed a target detection task so that we could ensure a relatively constant state of attentiveness. Trials containing target stimuli were excluded from all analyses.

The presentation of stimuli in the upper versus lower field was designed to help isolate the C1 wave, which is positive for lower-field stimuli and negative for upper-field stimuli as a result of the unique folding pattern of area V1 in the calcarine fissure (Clark et al., 1995, Jeffreys and Axford, 1972). No stimuli were presented in the 7.5° (polar angle) adjacent to the vertical meridian, or the 22.5° (polar angle) adjacent to the horizontal meridian of each quadrant (see Fig. 1) where there is likely to be substantial variation in individuals' C1 polarity (Clark et al., 1995). The remaining 60° of each quadrant were divided into 4 equal sectors, in which the center of each stimulus was presented at an eccentricity of 7.5° of visual angle. The simultaneous stimuli were presented either in adjacent sectors or were separated by a single sector.

Three different trial types were presented in random order (each occurring in 1/3 of trials): *dual-near* stimuli, in which two checkerboards were presented on each side simultaneously in adjacent sectors (0.16° visual angle gap, 1.88° center-to-center), *dual-far* stimuli, in which two checkerboards were presented on each side simultaneously separated by one sector (2° gap, 3.72° center-to-center), and *single* stimuli, in which one checkerboard was presented on each side. For each trial type, the target checkerboards appeared in each sector with equal probability, and stimuli were presented either in the upper or lower halves of the screen with equal probability. In the dual-near trials, the stimuli were separated by a gap of only 0.16° visual angle, which is much less than the size of a V1 RF at an eccentricity of 7.5° (approx. 0.6°; Hubel and Wiesel, 1974). Therefore, near stimuli should often fall into the same V1 RFs. In the dual-far trials, the inter-stimulus gap measured 2°, making two far stimuli too far apart to fall within the same V1 RF. However, the two dual-far stimuli should frequently fall within the same RF in extrastriate cortex. For example, V4 RFs are approximately 5° wide at an eccentricity of 7.5° (Gattass et al., 1988). In addition, the two dual-near stimuli should always compete in V4 RFs. Note that the classical RFs of neurons in V1 and V4 do not cross far into the ipsilateral side, so there should be minimal direct competition between the mirror-image stimulus pairs used in the present study.

### Procedure

Participants were presented with a sequence of 3840 stimulus displays, divided into 8 blocks. Each stimulus display was presented for a duration of 100 ms, separated by a blank interstimulus interval between 600 and 800 ms (rectangular distribution). Participants were instructed to press a key with the index finger of the right hand if a target was detected in any location and to withhold the response if a target was not detected. As an incentive to attend fully to the task, participants earned points for correctly identifying missing checks and lost points for incorrect responding, and those reaching the highest point total were awarded £10 online shopping vouchers.

### EEG acquisition/processing

**Fig. 3:**
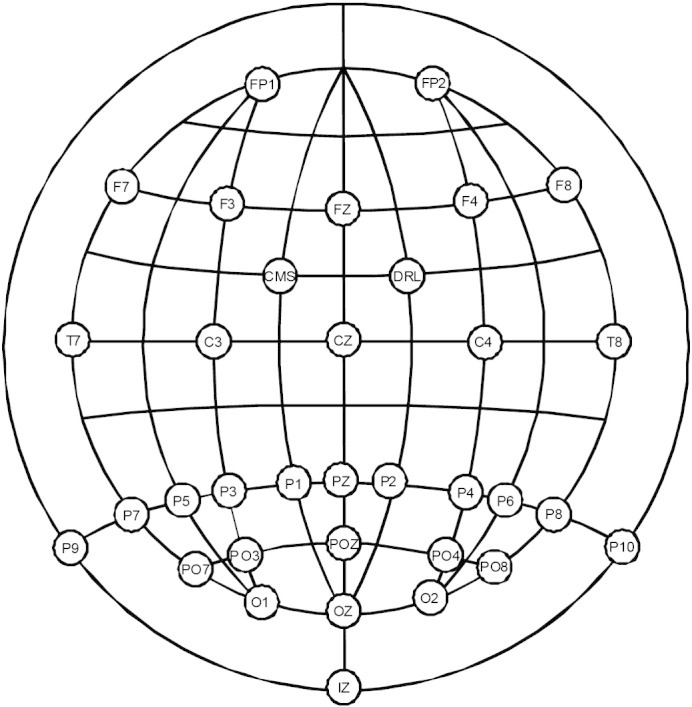
Electrode locations.

BioSemi ActiveTwo active Ag/AgCl electrodes (BioSemi, Amsterdam, Netherlands) were used to record the EEG. Thirty-two scalp electrodes were used, distributed across the whole head but with a higher density in parietal and occipital areas (see Fig. 3). EEG recordings were also taken from the left and right mastoids, and electrooculogram (EOG) recordings were taken from electrodes placed above and below the right eye and adjacent to the outer canthus of each eye. These signals were recorded in single-ended mode, low-pass filtered with a 5th-order sinc function (half-power cutoff at 410 Hz), and digitized at 2048 Hz. A photosensor was used to assess timing delays; all timings were within 2 ms, and there were no timing differences between the different stimulus locations (which is an advantage of our LCD display over a CRT display).

The data were processed with ERPLAB (Lopez-Calderon and Luck, 2014; http://www.erpinfo.org/erplab/) and EEGLAB (Delorme and Makeig, 2004; http://sccn.ucsd.edu/eeglab/), which are open source, Matlab-based toolboxes. The EEG and EOG signals were band-pass filtered using a noncausal Butterworth infinite impulse response filter with half-amplitude cut-offs of 0.5 and 30 Hz (12 dB/octave), down-sampled to 256 Hz, and re-referenced to the average of the mastoid electrodes. The data were epoched into 600-ms segments, including a 100-ms pre-stimulus baseline.

The EOG was referenced into bipolar horizontal and vertical derivations and used in the detection of eyeblinks and saccades. The analyses were limited to nontarget trials on which no response was made. In addition, trials were automatically rejected if a change in voltage exceeding 100 μV was detected in any channel (or 50 μV in the vertical EOG channel) within a moving window of 200 ms. Trials were also rejected if a step function detected changes of more than 50 μV in the vertical EOG channel or more than 10 μV on the horizontal EOG channel (Luck, 2014). The EEG was then visually examined for each participant, and thresholds were adjusted where necessary to ensure that all artefacts were rejected. In the final sample, an average of 13.65% of trials was rejected (SD = 7.43).

For each trial type, we averaged across all trials remaining after artefact rejection, collapsing over stimulus locations except that we kept the upper and lower visual field trials separate. The C1 component was isolated by subtracting the ERPs elicited by stimuli presented in the lower visual field from those elicited by stimuli in the upper visual field. This difference wave takes advantage of the fact that the upper and lower field representations in area V1 are located in the lower and upper banks of the calcarine fissure. The dipoles for the lower and upper fields therefore point in opposite directions, causing the scalp ERP to have opposite polarities for lower and upper field stimuli (Jeffreys and Axford, 1972, Clark et al., 1995). An upper-minus-lower difference wave therefore accentuates activity arising from V1, and activity that does not differ systematically for upper and lower visual field stimulation is cancelled out. Although other visual areas may also exhibit different activity for upper and lower field stimuli (Ales et al., 2010), the timing, scalp distribution, and precise reversal point of the C1 wave provide converging evidence that it arises from area V1 (Clark et al., 1995, Kelly et al., 2013a). For the P1 wave, we averaged across upper and lower visual field stimuli.

### Statistical analyses

In analyzing the data, we compared the waveforms elicited by dual-near and by dual-far trials with both the average of the two single-stimulus waveforms (*single averages*) and with the sum of the two single-stimulus waveforms (*single sum* averages). As illustrated in Fig. 2, the single sum waveforms provide an estimate of the response that would be obtained if the two simultaneous stimuli were processed completely independently, with no competition, and the single average waveforms provide an estimate of what might be expected if the two stimuli strongly compete (Reynolds et al., 1999). For each component, statistical analyses were conducted to compare the following trial types: dual-near vs. dual-far stimuli, both dual-near and dual-far vs. single average, and dual-near and dual-far vs. single sum.

We created two a priori clusters of electrodes, one for the C1 and C2 waves and one for the P1 wave. Following from previous research, the C1/C2 cluster included the electrodes at or near the posterior midline (P1, O1, Iz, Pz, P2, POz, Oz, O2). The remaining posterior sites were used for the P1 cluster (P3, P5, P7, P9, PO7, PO3, P4, P6, P8, P10, PO8, PO4). The ERP waveforms were averaged across the sites within a cluster prior to quantification of component amplitudes.

We chose our time windows with the main aim of minimizing the influence of overlap from other components on our components of interest. For the C1 and C2 we used the signed area approach (Sawaki et al., 2012, Luck, 2014), in which the area either above or below the baseline is measured within the specified time window (with points of the opposite polarity effectively set to zero). This approach is ideal for components measured from a difference wave because it is not necessary to define a narrow measurement window to avoid cancellation of one component by an opposite-polarity component. We chose wide windows within which each component of interest has been found in previous studies. Specifically, we used the negative area between 50 and 150 ms for the C1 wave (Di Russo et al., 2002, Jeffreys and Axford, 1972, Fu et al., 2010) and the positive area between 50 and 250 ms for the C2 wave (Fortune and Hood, 2003, Kappenman and Luck, 2012). The signed area method was unsuitable for the P1 because it was not measured from a difference wave. We instead measured the mean amplitude within a specific time window. To avoid biasing the results by using differences between conditions to define the time window, we collapsed across conditions and then defined the time window as the time between P1 onset and P1 peak (thereby minimizing overlap from the N1 wave). Specifically, we computed a grand average across all trial types and used the 20% fractional peak latency to define P1 onset latency (Kiesel et al., 2008) and the time of the maximum positive voltage to define P1 peak latency. This resulted in a measurement window of 78–117 ms.

Statistical analyses were conducted with analysis of variance (ANOVA), and all *p* values presented below reflect the Huynh-Feldt correction for heterogeneity of covariance.

### Source analyses

To obtain information about the possible neural sources of the data, we applied low resolution electromagnetic tomography (sLORETA; Pascual-Marqui, 2002) to the C1, C2, and P1 measurements, using a cortical surface reconstruction of the standard MNI152 brain to constrain the solutions. These analyses were performed on the grand average data to maximize the signal-to-noise ratio. Note that the goal of these analyses was not to provide definitive evidence regarding the neural generators, but simply to determine whether the scalp distributions were at least consistent with the assumed generator locations of the C1, C2, and P1 waves.

## Results

### C1 analyses

**Fig. 4:**
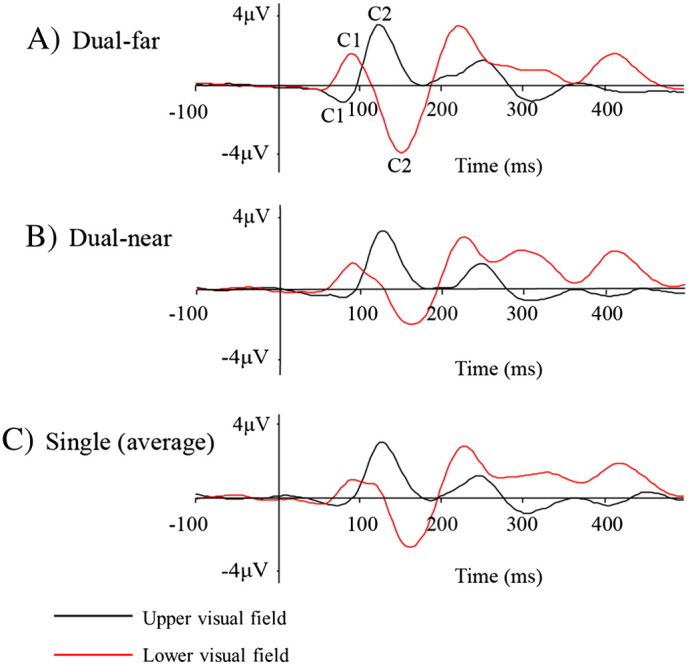
Grand average ERP waveforms from the C1/C2 electrode cluster (P1, O1, Iz, Pz, P2, POz, Oz, O2, referenced to the averaged mastoids) for upper- and lower-field stimuli in each of the stimulus configurations.

**Fig. 5:**
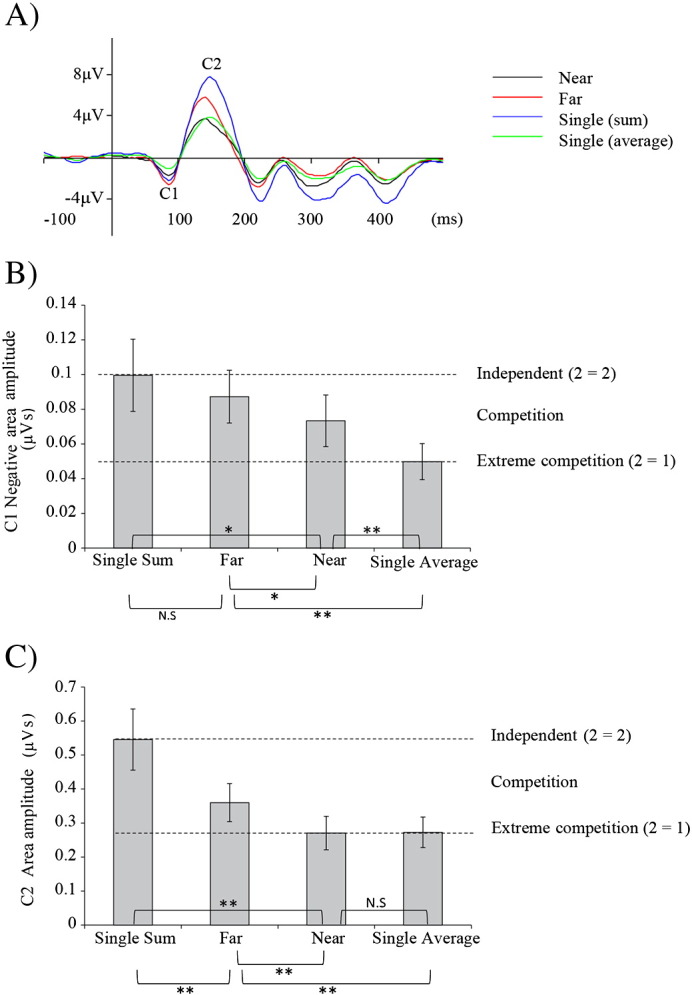
A) Grand average upper-minus-lower difference waves from the C1/C2 electrode cluster (P1, O1, Iz, Pz, P2, POz, Oz, O2, referenced to the averaged mastoids) in each of the stimulus configurations. B) Area of the negative region in the C1 time window for the 4 trial types, with pairwise significance levels and error bars representing the standard error of the mean. *p < .05. **p < .01. C) Area of the positive region in the C2 time window for the 4 trial types, with pairwise significance levels and error bars representing the standard error of the mean. *p < .05. **p < .01.

As shown in Fig. 4, the average activation measured by electrodes in the posterior midline cluster was initially more negative for stimuli presented in the upper field and more positive for stimuli presented in the lower field. This difference was present from approximately 60–100 ms, and then the polarities reversed. The initial upper-lower difference is the C1 wave, which can be seen even more clearly in the upper-lower difference waves shown in Fig. 5A. The C1 in the grand average was smallest in response to the single stimuli (when averaged rather than summed), was larger for the dual-near stimuli, and was even larger for the dual-far stimuli. The C1 for the dual-far stimuli was similar in size to that of the single sum waveform, suggesting that the far stimuli were processed largely independently at this stage. In contrast, the C1 for the dual-near stimuli was approximately midway between the C1 for the single sum and single average stimuli, consistent with competition between the two dual-near locations.

**Fig. 6:**
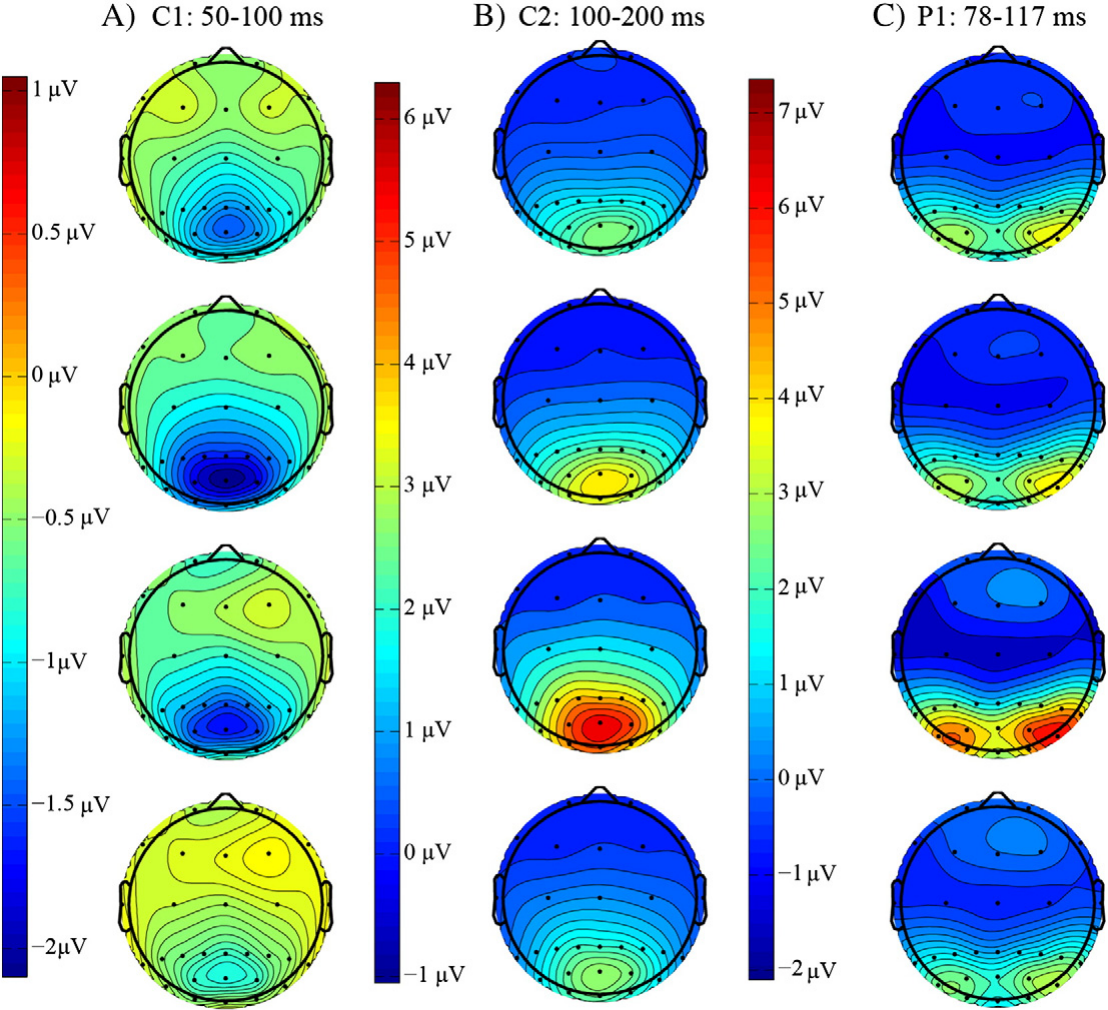
A) C1 and B) C2 scalp maps, showing the distribution of voltage in the upper minus lower difference waves from 50 to 100 ms, and 100 to 200 ms, respectively. C) P1 scalp maps, showing the distribution of voltage in the time range of the early portion of the P1 wave.

As shown in Fig. 5A, the C1 was followed by an opposite-polarity difference between the upper and lower fields (the C2 wave). The timing of the C2 wave is consistent with the timing of feedback into V1 from extrastriate areas (e.g., Fahrenfort, Scholte & Lamme, 2008), suggesting that the C2 reflects feedback signals arriving into V1 (although we cannot be certain of this). Figs. 6A and B show the scalp distributions of the upper-minus-lower difference waves from 50 to 100 ms (C1) and 100 to 200 ms (C2) respectively. Both of these scalp distributions exhibited a focus over the occipital pole, which is consistent with a generator in area V1 (but does not rule out other potential generators, such as V2).

The C1 amplitude measurements for dual-near, dual-far and single average conditions were submitted to a one-factor repeated measures ANOVA, which revealed a significant main effect, *F*(2, 32) = 12.67, *p* < .001. Planned two-tailed paired-samples t-tests were then conducted to test specific hypotheses (see Fig. 5B). First, we tested the hypothesis that greater competition would occur between simultaneous stimuli when the gap between the stimuli was small, by comparing the dual-near and dual-far trial types. We found that the C1 was significantly smaller for the dual-near stimuli than for the dual-far stimuli, *t*(16) = − 2.41, *p* = .028, confirming this prediction.

Next, to determine the level of competition between the dual-far stimuli, we compared the C1 in this trial type with the single sum and single average C1 waveforms. The C1 in the dual-far waveforms was nearly identical to the C1 in the single sum waveforms, *t*(16) = − 1.07, *p* = .299, which is what would be expected if the two stimuli in the dual-far trials were processed completely independently. Consistent with this, the C1 for the dual-far trials was significantly greater than the single average C1, *t*(16) = 4.38, *p* < .001.

The same comparisons were performed for the dual-near stimuli. The dual-near stimuli elicited significantly less activation than single sum, *t*(16) = − 2.38, *p* = .030, providing further evidence for competition between the near stimuli. The C1 to dual-near stimuli was also significantly greater in amplitude than the single average C1, *t*(16) = − 2.96, *p* = .009,^1^ suggesting that two near stimuli did not compete to the extent of producing the same activation as a single stimulus.

**Fig. 7:**
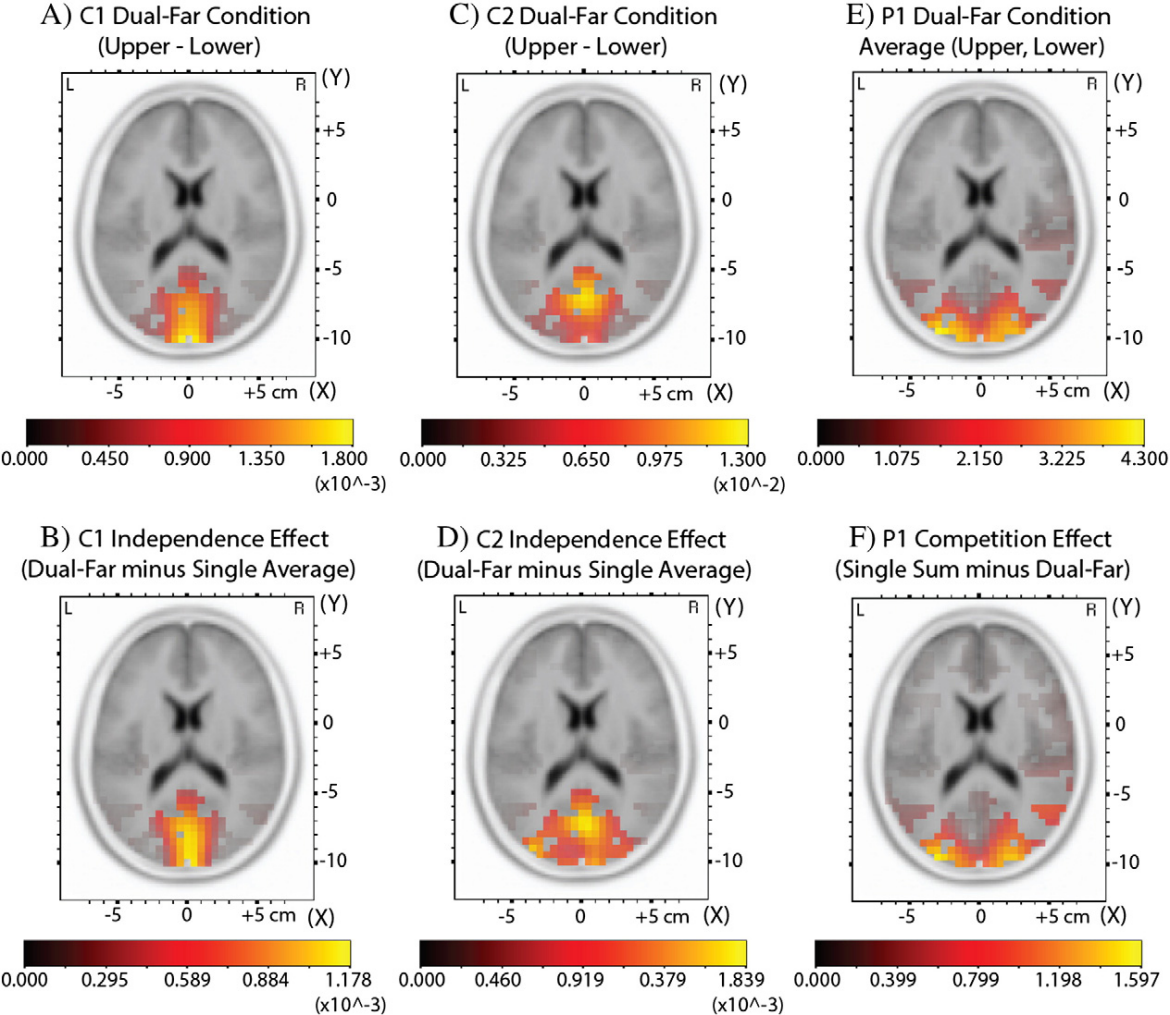
Estimated distribution of current flow over the cortical surface for A) Dual-Far upper-minus-lower difference wave, B) C1 Dual-Far difference wave minus Single Average difference wave, C) C2 Dual-Far upper-minus-lower difference wave, D) C2 Dual-Far difference wave minus Single Average difference wave, E) P1 Dual-Far average, F) P1 Single Sum average minus Dual-Far average. Note that each map has a separate scale to maximize the visibility of the estimated current distribution.

Fig. 7A shows the sLORETA solution for the C1 wave, based on the upper-minus-lower difference waves for the dual-far trials, for which the C1 was largest (solutions were similar for the other trial types). The estimated current flow was maximal along the occipital midline, which is the location of area V1. The Talairach coordinate of the maximum current flow was (X = − 5, Y = − 96, Z = 19), which is similar to the average coordinates of the C1 dipoles (X = 1, Y = − 85, Z = 12) reported by Di Russo et al. (2002), which in turn closely matched the location of area V1 determined from fMRI data. Note that some differences in V1 coordinates would be expected owing to differences in stimulus location between studies.

We also conducted an sLORETA analysis on a difference wave that focused on the independence of the C1 when processing two simultaneous stimuli in the same hemifield. Specifically, we took the upper-minus-lower difference waves and subtracted the single average condition from the dual-far condition. Any difference between these two upper-minus-lower difference waves reflects independent (additive) contributions of the stimuli in the dual-far displays. Fig. 7B presents the sLORETA solution for this double difference wave, showing that—like the basic C1 itself—the estimated current flow for this “independence effect” is maximal along the occipital midline. The present data do not provide the precision needed to distinguish between area V1 and the surrounding extrastriate areas, but these results demonstrate that the scalp distributions of the C1 wave and the C1 independence effect are at least consistent with a generator in area V1.

### C2 analyses

For the C2 wave, a repeated measures ANOVA between dual-near, dual-far and single average trial types again revealed a significant main effect, *F*(2, 32) = 7.85, *p* = .002. The follow-up t-test between the dual-near and dual-far trial types was significant, *t*(16) = − 3.98, *p* = .001, once again demonstrating significantly greater competition between the dual-near stimuli than between the dual-far (Fig. 5C).

However, for both the dual-near and dual-far stimuli, the C2 wave showed greater evidence of competition between the simultaneous stimuli than was observed for the C1 wave. This can be seen by comparing Figs. 5B and C, in which the amplitudes for the simultaneous stimuli were closer to the “extreme competition” level for the C2 wave than for the C1 wave. This greater degree of competition was also evidenced in the statistical comparisons. Paired t-tests indicated significant differences in C2 amplitude between the dual-far and single sum waveforms, *t*(16) = − 3.79, *p* = .002, and between the dual-far and single average waveforms, *t*(16) = 3.20, *p* = .006. These effects indicate a moderate level of competition between the far locations at the C2 stage. Paired t-tests also yielded a significant difference between the dual-near and single sum trial types, *t*(16) = − 4.95, *p* < .001, but not between the dual-near and single average trials, *t*(16) = − 0.82, *p* = .936. These effects suggest fairly extreme competition between the two locations in the dual-near stimuli at the C2 stage.

The sLORETA solutions for the C2 wave (based on the upper-minus-lower difference wave for the dual-far trials) and the C2 independence effect (dual-far minus single average) are shown in Figs. 7C and D. Like the C1 solutions, the maximal current flow was along the occipital midline, consistent with a generator in area V1. However, the C2 solutions were more broadly distributed, suggesting that the C2 wave and C2 independence effect may involve the surrounding extrastriate areas as well as striate cortex.

The Talairach coordinate of the maximum current flow for the C2 wave was (X = 0, Y = − 67, Z = 26), which is reasonably close to the coordinate of the maximum current flow for the C1 component, as described above. Moreover, it was right on the midline rather than being on the lateral surface of the brain, which is also consistent with a location in area V1 (or in areas V2 or V3).

### P1 analyses

**Fig. 8:**
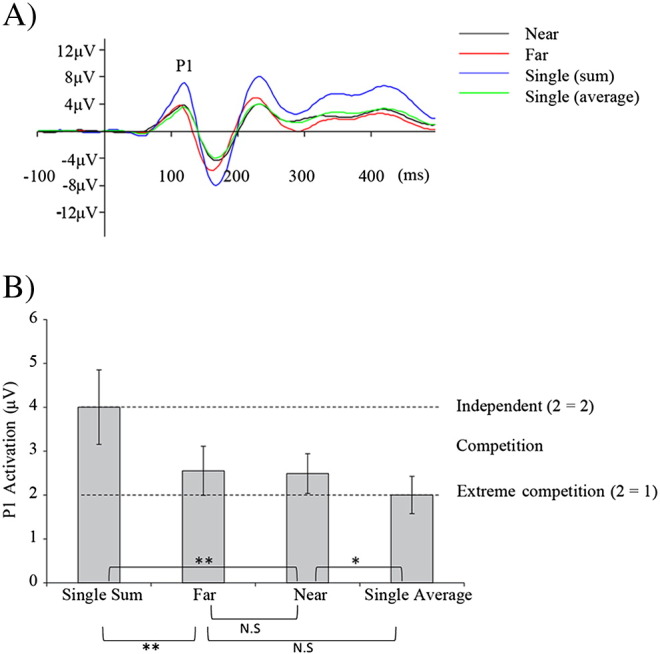
A) Grand average ERP waveforms from the P1 electrode cluster (P3, P5, P7, P9, PO7, PO3, P4, P6, P8, P10, PO8, PO4, referenced to the averaged mastoids), collapsed across upper- and lower-field locations in each of the stimulus configurations. B) Mean amplitude in the P1 time window for the 4 trial types, with pairwise significance levels and error bars representing the standard error of the mean. *p < .05. **p < .01.

The P1 wave can be easily observed in the waveforms from the lateral occipital electrode sites, collapsed across upper and lower fields (Fig. 8A). Scalp maps for the individual conditions (Fig. 6C) also show bilateral lateral occipital activation in all conditions. P1 amplitude differed only slightly among the dual-near, dual-far, and single average waveforms, but it was much smaller for these waveforms than for the single sum waveforms. This suggests that competition between the two simultaneous stimuli was fairly substantial.

A repeated measures ANOVA between dual-near, dual-far and single average trial types revealed a marginally significant main effect, *F*(2, 32) = 3.67, *p* = .050. Planned paired-samples t-tests (Fig. 8B) yielded significant differences between the dual-near and single sum waveforms, *t*(16) = − 3.18, *p* = .006, and also between the dual-far and single sum waveforms, *t*(16) = − 3.17, *p* = .006. This indicates that competition was present between the two stimulus locations in both the dual-near and dual-far trials. In addition, the dual-near and dual-far P1 waves were nearly identical, with no significant difference between them, *t*(16) = − 0.34, *p* = .737. This suggests that competition is equivalent between these two distances at the P1 stage. The P1 was also significantly larger for the dual-near waveform than for the single average waveform, *t*(16) = 2.61, *p* = .019 indicating that activation for the near stimuli was not reduced to the level of a single stimulus. The magnitude of this difference was nearly identical when the dual-far waveforms were compared with the single average waveforms, but this difference did not quite reach significance, *t*(16) = 1.95, *p* = .070. This small difference in *p* values presumably reflects noise rather than any real difference between the dual-near and dual-far conditions. In any case, the pattern of results clearly indicates that the two locations compete with each other at the stage of the P1 wave in both dual-near and dual-far configurations, but it is likely that the competition does not bring the P1 amplitude all the way down to the level observed for a single stimulus.

For the P1 wave, sLORETA was applied to the data from the dual-far trials after averaging across upper- and lower-field stimuli. As shown in Fig. 7E, the estimated distribution of current flow was more lateral for the P1 wave than for the C1 and C2 waves. sLORETA was also applied to the difference in P1 amplitude between the single sum waveforms and the dual-far waveforms, which is a means of quantifying the effect of competition on the P1 wave. In other words, this amplitude increases as the dual-far waveform becomes less and less like the sum of the activity to the individual stimuli. As shown in Fig. 7F, the estimated current flow for this “competition effect” was also maximal over lateral occipital cortex.

The maximal current flow for the P1 wave had coordinates of (X = − 20, Y = − 97, Z = 5) in the left hemisphere and (X = 15, Y = − 97, Z = 5) in the right hemisphere. These are fairly close to the average coordinates of the early P1 dipole (X = ± 32, Y = − 84, Z = 10) identified by Di Russo et al. (2002), which was localized on the basis of fMRI-based topographical mapping to areas V3/V3a and surrounding areas of the middle occipital gyrus. Again, it should be noted that some differences in coordinates would be expected between studies due to differences in stimulus location. Thus, we cannot say that the P1 in the present study had exactly the same generator as the P1 in the Di Russo et al. (2002) study, but the two studies yielded reasonably similar estimates.

## Discussion

Overall, the pattern of results from this experiment indicates that the spatial range of competition between simultaneous stimuli increases between the initial C1 wave (which likely reflects V1 neurons) and the P1 wave (which likely reflects neurons in extrastriate cortex). This pattern is consistent with decades of single cell neurophysiological research (e.g., Luck et al., 1997, Moran and Desimone, 1985, Reynolds et al., 1999, Treue and Maunsell, 1996) as well as human fMRI research (Kastner et al., 1998, Kastner et al., 2001), and it demonstrates that the present experimental approach provides a sensitive and valid means of assessing competitive interactions at specific time points and inter-item distances. This approach therefore provides a valuable tool that can be used to assess how these interactions vary as a function of stimulus properties (e.g., similarity) and cognitive manipulations (e.g., attention) in human subjects. An analogous approach has been used in fMRI experiments (e.g., Kastner et al., 1998, Kastner et al., 2001), but the present approach can isolate competition at specific time points rather than collapsing across hundreds or thousands of milliseconds.

We found that the C1 response to two simultaneous stimuli was nearly equivalent to the sum of the C1 responses to the two individual stimuli when they were separated by a 2° gap, indicating minimal competition at this distance in primary visual cortex. However, when the separation was reduced to 0.16°, the C1 response to the two simultaneous stimuli was significantly less than the response to the two stimuli presented individually (and smaller than the response to two stimuli presented simultaneously with a 2° gap). This provides strong evidence of competition between the two stimuli at this distance. However, the C1 response to the dual-near stimuli was still larger than the response to a single stimulus, indicating that the competition was only moderate. This likely reflects the fact that, although some V1 receptive fields contained parts of both stimuli, many other V1 receptive fields presumably contained just one of the two stimuli.

It is important to note that there has been some recent controversy about whether the C1 wave necessarily reflects activity in area V1 (Ales et al., 2010, Ales et al., 2013, Kelly et al., 2013a, Kelly et al., 2013b). The most plausible conclusion from these papers is that a polarity reversal for upper- versus lower-field stimuli is not by itself sufficient to discriminate among V1, V2, and V3, but the combination of the particular point at which the polarity reverses (Clark et al., 1995, Jeffreys and Axford, 1972) and the scalp distribution of the C1 provide strong evidence that it arises from area V1. Thus, the present C1 results very likely reflect V1 with little or no contribution from V2 and V3.

The pattern of results was quite different for the P1 wave, which likely arises from extrastriate cortex (Di Russo et al., 2002). Pairs of stimuli produced similar P1 amplitudes regardless of the inter-item distance, and the amplitudes for these pairs were substantially reduced relative to the sum of the single-stimulus P1 responses. The competition brought the P1 amplitude for the pairs most of the way (but not all of the way) to the level of the response to a single stimulus. These results indicate the presence of substantial and approximately equivalent competition between locations in dual-near and dual-far trials. Note, however, that the distance between stimuli was only 2° in the dual-far trials, and the amount of competition would likely be reduced if the distance between the stimuli were increased further.

The C2 component showed a pattern than was different from both the C1 and P1 components. Less is known about this component, but the fact that its polarity reverses for upper versus lower field locations suggests that it may arise in area V1 (and also perhaps V2 and V3), and its timing suggests that it reflects feedback from later visual areas. Its scalp distribution is also consistent with a generator in striate and/or early extrastriate areas (Fig. 6B). Like the C1 wave, the C2 wave was smaller for dual-near stimuli than for dual-far stimuli, indicating that competition can be reduced by a spatial gap of only 2° at this stage. However, like the P1 wave, the C2 wave was significantly smaller for dual-far stimuli than for the sum of two single-location stimuli, indicating that at least some competition was present even with a 2° gap between the stimuli. This pattern may reflect the combination of an anatomically early generator source (V1 and perhaps V2/V3) along with a relatively late time of occurrence (after the P1 wave). This combination may allow differential competition between our near and far stimulus pairs (separated by a 0.16° gap versus a 2° gap) owing to the small receptive fields in area V1, while still providing substantial interactions between the stimuli owing to feedback from areas with larger receptive fields. However, additional research is needed to determine the anatomical generator of the C2 wave and to assess the hypothesis that it reflects feedback from higher-level extrastriate areas into striate and early extrastriate areas.

Although competition in primate visual cortex has previously been investigated using single cell recordings (e.g., Moran and Desimone, 1985, Luck et al., 1997, Chelazzi et al., 1998, Reynolds et al., 1999), V1 cannot easily be investigated using this approach due to its extremely small RF sizes. In contrast and underscoring the importance of the present findings, the ERP approach allows measurement of activation of neural populations in human visual cortex with excellent temporal resolution. Previous studies by Kastner et al. (1998) found BOLD activation in V1 and extrastriate cortex to be modulated by manipulating the extent to which stimuli compete within a RF. To our knowledge, ours is the first study of human brain activity to demonstrate that this competition begins in the initial, presumably feedforward, V1 response. Further competition for items of both high and low proximity appears to be enabled by feedback into V1 from extrastriate areas, which have RFs representing a larger proportion of the visual field (Kastner et al., 2001).

Inter-stimulus competition plays an important role in many perceptual and cognitive processes, including crowding, visual attention (e.g., Desimone and Duncan, 1995) and visual short-term memory (e.g., Shapiro and Miller, 2011). For example, Lavie's perceptual load theory proposes that attention can operate at an early stage only when competition produces a high perceptual load (Lavie, 1995, Lavie, 2005). However, studies of this sort typically lack an independent measure of the degree of competition between the stimuli. The approach outlined in this study will therefore be useful in testing specific predictions of how, and at which stage in processing, competition influences these perceptual and cognitive processes.

## Footnotes

1 All of the planned comparisons that were significant in this study remained significant after False Discovery Rate correction for multiple comparisons.
